# Parenting Styles, Internalization of Values and Self-Esteem: A Cross-Cultural Study in Spain, Portugal and Brazil

**DOI:** 10.3390/ijerph17072370

**Published:** 2020-03-31

**Authors:** Isabel Martinez, Fernando Garcia, Feliciano Veiga, Oscar F. Garcia, Yara Rodrigues, Emilia Serra

**Affiliations:** 1 Department of Psychology—Social Psychology Area, University of Castilla-La Mancha, Avda de los Alfares 44, 16071 Cuenca, Spain; 2 Department of Methodology of the Behavioral Sciences, University of Valencia, Av. Blasco Ibanez, 21, 46010 Valencia, Spain; fernando.garcia@uv.es; 3 Instituto de Educação, Alameda da Universidade, Universidade de Lisboa, 1649-013 Lisboa, Portugal; fhveiga@ie.ulisboa.pt; 4 Department of Developmental and Educational Psychology, University of Valencia, Av. Blasco Ibanez, 21, 46010 Valencia, Spain; oscar.f.garcia@uv.es (O.F.G.); emilia.serra@uv.es (E.S.); 5 Department of Education, Faculdade São Braz, Curitiba, Parana, 82520-590, Brazil; yaraiglesia@gmail.com

**Keywords:** parenting, parental warmth, parental strictness, self-esteem, social values, culture

## Abstract

The present study analyzes the impact of parenting styles on adolescents’ self-esteem and internalization of social values in three countries, Spain, Portugal and Brazil. The sample of the study was comprised of 2091 adolescents from Spain (*n* = 793), Portugal (*n* = 675), and Brazil (*n* = 623) from 12–18 years old (52.1% females). The four types of parenting styles, authoritative, indulgent, authoritarian and neglectful, were measured through the warmth and strictness dimensions of the Scale of Parental Socialization ESPA29. The two criteria variables were captured with the five dimensions of the AF5, Five-Factor Self-Concept Questionnaire, and with self-transcendence and conservation Schwartz values. Results confirm emergent research in parenting socialization: the use of parental warmth is evidenced as key for adolescent self-esteem and internalization of social values in the three countries analyzed. Indulgent and authoritative parenting (both characterized by parental warmth) are associated with the highest value internalization in the three countries. Furthermore, indulgent parenting (use of warmth) is associated with the highest adolescent self-esteem, overcoming authoritative parenting (use of warmth and strictness). The influence of parenting over adolescent self-esteem and values internalization is maintained independent of the differences in self-esteem and value priorities observed in the cultural context, the sex and age of the participants.

## 1. Introduction

Internalization of social values, which refers to the assumption that society is one’s own so that socially acceptable behavior is motivated by internal rather than external factors, is one of the main objectives of parental socialization (Grusec & Goodnow, 1994) [1] (p. 4), and has been identified by earlier scholars as the key to raise well-developed children [2,3,4,5,6]. It has also been noted that the specific parenting practices used be parents differentially affect children’s internalization of values [1,5,7].

Swarchtz values theory has recently identified conservation and self-transcendence values as social focused values [8], since these values are centered on consideration for others and acceptance of social norms [5]. Self-transcendence values express concern for the welfare and interests of others and include two types of values: universalism and benevolence [9,10,11,12]. Universalism is described as “understanding, and protection for the welfare of all and the environment” [11] (p. 4), whereas benevolence is described as “preservation and enhancement of the welfare of people with whom one is in frequent personal contact” [11] (p. 4). Conservation values refer to the respect, commitment and acceptance of the customs and ideas that traditional culture or religion provide the self [9,11]. Conservation values include three types of value priorities: Security, conformity and tradition [8,10,13]. Security values are described as “search of safety, harmony and stability of society, of relationships, and of self” [11] (p. 3); conformity values refer to “restraint of actions likely to upset others and violate social expectations or norms” [11] (p. 3); and tradition values are described as “respect, commitment and acceptance of the customs and ideas that traditional culture or religion provide the self” [11] (p. 4). Self-transcendence and conservation have previously used to measure social values internalization [5,6,14,15], following Grusec and Goodnow’s (1994) [1] (p. 4) suggestion of measure internalization in terms of prosocial behavior consideration for the feelings or needs of others—and moral standards—assessed, for instance, by resistance to temptation, reparation after deviation, evidence of guilt, and level of moral reasoning.

Despite the importance of the social focus in the socialization process, the individual focus is also essential in parental socialization. In this sense, self-esteem is also considered a main goal of parental socialization, being a classical criterion of adolescents’ internal well-being in parenting studies. Self-esteem, as the person’s perception of himself, has been considered key in understanding behavioral, cognitive, emotional, and social functioning [16]. Research has shown that self-esteem is related to a wide range of both psychological and behavioral criteria [17,18,19]. Finally, in the same way that values internalization, self-esteem has been proven to be differentially influenced by parental styles [20,21,22,23].

Research on parental socialization have traditionally considered two main independent dimensions to capture parenting styles. These two dimensions have been identified as parental warmth and parental strictness [24,25,26,27], although other different labels with similar meanings have been used [28,29]. The strictness dimension refers to the extent to which parents use control and supervision, establish norms for children’s behavior, and maintain position of authority [24,30,31]. Demandingness, control or firmness are some of the labels that also have been used to identify strictness dimension [24,26,32]. The warmth dimension represents the extent to which the parents show the children love and affection, give them their support, talk and reason with them [6,27,33]. The warmth dimension has been labeled as responsiveness, involvement, acceptance or implication [24,30,34]. Based on the use that parents do of the practices that define these two dimensions, four parenting styles are identified: authoritative parenting (characterized by the use of warmth and strictness), authoritarian parenting (mainly characterized by the use of strictness), indulgent parenting (mainly characterized by the use of warmth) and neglectful parenting (characterized by the lack of both warmth and strictness).

In relation to the four parenting styles and their effects on adolescent behavioral and psychological adjustment, finding out which is the optimal socialization style (i.e., authoritative, indulgent, authoritarian or neglectful) that parents should use has been the main goal of parenting studies [24]. Recent studies (see Garcia, Serra, Garcia, Martinez, & Cruise, 2019) [35] have proposed a paradigm of three different historical stages for optimal parental style. This new paradigm highlights that over the past century, based on numerous studies mainly carried in the United States, scholars as Baumrind [3,36,37,38] or Steinberg and colleagues [39,40,41,42] have pointed out authoritative parenting as the optimal parenting style (i.e., second stage) [35]. Authoritarian parenting (i.e., first stage) [35] was considered optimal at the beginning of the century by scholars such as Watson, (1928) [43], and has shown benefits among some ethnic minorities such as Asian–Americans [44,45], African Americans [46,47] or Arab families [48,49,50]. Finally, in the current digital era, emergent research has begun to point to indulgent parenting as the optimal style (i.e., third stage) [35]. Studies carried out in different cultural contexts have begun to show that indulgent parenting (characterized by the use of warmth but not parental strictness) leads to the same or higher offspring’s personal and social adjustment than authoritative parenting (characterized by the use of warmth and also by parental strictness) [35,51,52,53,54,55,56]. These studies have initially been conducted in European and Latin American countries, including Spain, Brazil and Portugal [6,22,57], although recently similar results have been found in countries such Sweden, Slovenia, the Czech Republic, the United Kingdom, the United States, Germany, Italy, Turkey or Norway [35,51,54,55,56,57]. Parenting styles seem to have a different impact depending on the child’s cultural background, in which parental socialization takes place [24,26,44,58,59], and recent research seems to suggest that the use of strictness is beginning to be perceived as unnecessary in parental socialization in an increasing number of different cultural contexts.

### The Present Study

This study analyzes adolescents’ self-esteem and value priorities in three countries, Spain, Brazil and Portugal. Furthermore, the impact of parenting styles on adolescents’ self-esteem and internalization of values (self-transcendence and conservation Schwartz values) [10] is also analyzed, contrasting the three cultures. Since recent research in these (and other European and Latin American) countries points out the indulgent style as optimal for adolescent adjustment [26,27,60], we expect that, despite the differences in value priorities and levels of self-esteem between adolescents from the three countries, indulgent parenting will prove to be the optimal parenting style in Spain, Portugal and Brazil. We hypothesize that indulgent parenting will be associated with higher or similar self-esteem and values internalization than authoritative parenting in the three cultures analyzed.

## 2. Materials and Methods 

### 2.1. Participants

The sample was composed of 2091 offspring adolescents (52.1% of them were women) covering the age range from 12–18 years old, M (Mean) = 14.28, SD (Standard Deviation) = 1.74. There were 1267 (60.6%) in early adolescence, from 12–14 years old, and 824 (39.4%) in late adolescence, from 15–18 years old, sampled from Spain (793, 37.9%; 56.4% being women; mean age = 14.47, SD = 1.73, range = 12–18 years; 443, 55.9%, being early adolescents), Portugal (675, 32.3%; 56.4% being women; mean age = 14.08, SD = 1.78, range = 12–18 years; 445, 65.9%, being early adolescents), and Brazil (623, 29.8%; 47.4% being women; mean age = 14.23, SD = 1.68, range = 12–18 years; 379, 60.8%, being early adolescents) (see Table 1).

### 2.2. Procedure

The sample frame of the present study were adolescents from secondary schools in large metropolitan areas (with over one million inhabitants in each area) on the East Coast of Spain, Middle Coast of Portugal, and in Southeast Brazil. The data was collected from 34 educational centers (11 Spanish, 13 Portuguese, and 10 Brazilian) selected through a simple random sampling method from a complete list of centers [19,26,27,61,62]. In the samples of the three countries, we selected adolescents from middle class neighborhoods who: (a) lived in two-parent nuclear families, mother or primary female caregiver and father or primary male caregiver; and (b) whose parents and four grandparents were born in the country of each sample (Spain, Portugal and Brazil).

The minimum sample size required for conventional statistical errors type I, α = 0.05, and type II, β = 0.05, was calculated with a priori power analysis [63,64], it was fixed at a medium-small effect size (*f* = 0.17, estimated from ANOVAs (Analysis of Variance) by Lamborn et al., 1991 [41]) in an univariate F-test between the four parenting style groups [27,65]. A priori power analyses (α = 0.05, 1 – β = 0.95, and *f* = 0.17) showed a minimum sample size of 600 participants [35,51]. In the three sampled countries, the sample size was always over the minimum sample size required. A post-hoc power analysis [63,64] showed that the F test could detect, in the worst situation (i.e., Brazil, *N* = 623, α = β = 0.05), the expected effect size (*f* = 0.17), with a power that exceeded the a priori fixed value (1 − β = 0.96). Additionally, the sensitivity power analysis with the full sample (*N* = 2091, α = β = 0.05) showed that *F* main effects between the four parenting styles can detect even a small effect size (*f* = 0.09) [63,64,66]. The nearest 5% (*n* = 121) of the cases contained these inconsistencies and were deleted from the study sample. All of the questionnaires were completed anonymously following Institutional Review Board approval, 10 March 2017.

### 2.3. Instruments

#### 2.3.1. Parental Socialization

Parental Socialization styles were measured with the families’ acceptance/involvement and strictness/imposition axe dimensions (ESPA29, Parental Socialization Scale [67]). A self-report instrument was designed to measure the parental socialization styles through children and adolescents’ responses. The family acceptance/involvement dimension was measured with the paternal and maternal practices of warmth (“He/she shows affection”), reasoning (“He/she talks to me”), indifference (“He/she seems indifferent”), and detachment (“It’s the same to him/her”). Parental practices of detachment and indifference have a negative relation to the acceptance/involvement dimension. The family strictness/imposition dimension was measured with the paternal and maternal practices of revoking privileges (“He/she takes something away from me”), verbal scolding (“He/she scolds me”), and physical punishment (“He/she hits me”). All parental practices were measured in 29 appropriate contexts, with 13 scenarios which sample contexts of obedience where the family norm is followed by the child (e.g., “If somebody comes over to visit and I behave nicely”), and in 16 scenarios which sampled the context of disobedience where the family norm is contravened by the child (e.g., “If I have broken or spoiled something”). The adolescent responded with a four-point scale to indicate the frequency in which their father and mother make use of the seven specified parental practices, with a range from one (“never”) to four (“always”).

The ESPA29 factor structure has been widely analyzed with exploratory [67,68,69] and confirmatory analyses [28,29]. The instrument was firstly developed and normalized in Spain [67] and translated to English [29,35], German [35], Portuguese [28], Brazilian-Portuguese [68,69], and Basque [70] languages. ESPA 29 have used it to validate other parenting instruments [71] analyzed by meta-analysis studies [58,72] and to examine maternal and paternal contributions to family socialization [33]. The ESPA29 styles, dimensions and practices are related to multiple socialization outcomes such as bullying [27,73] and cyber-bullying [27,74,75,76], hostility [77], child-to-parent violence [53,78], reactive and proactive adolescent violence [52], dating violence [79], drug use [80,81,82], adolescent behavior problems [83], empathy and connectedness with nature [84], self-concept [35,57], and prosocial values during parenting socialization [6,35]. Cronbach’s alphas in this study for the two main dimensions were acceptance/involvement, 0.971, and strictness/imposition, 0.960. For each subscale, they were warmth, 0.960, indifference, 0.944, reasoning, 0.951, detachment, 0.917, verbal scolding, 0.938, physical punishment, 0.952, and revoking privileges, 0.951.

#### 2.3.2. Multidimensional Self-Concept

Self-concept was measured with the AF5’s Five-Factor Self-Concept dimensions [85], academic (e.g., “I do my homework well”), social (e.g., reversed item, “It is difficult for me to talk to strangers”), emotional (e.g., reversed item, “Many things make me nervous”), family (e.g., “My family would help me with any type of problem”), and physical (e.g., “I take good care of my physical health”). This multidimensional self-concept scale has 30 items exactly divided into six items by dimension. The participant rates each item according to his/her level of agreement or disagreement using a 99-point scale (depicted which a thermometer), which ranges from one, representing full disagreement, to 99, representing full agreement. Modifications were made in each dimension’s scale to obtain a score which ranged from 0.10–9.99.

The five-factor multidimensional structure of the AF5 was analyzed with exploratory [85] and confirmatory [18,86,87] analyses, and none appear to be related to method effects among negatively worded items [88,89]. The instrument was originally developed and normalized in Spain [85] and it has been translated to English [90], Portuguese [91], Brazilian-Portuguese [18], Basque [92], and Catalan [93] languages. AF5 dimensions have applied in multiple research fields such as behavior problem profiles during adolescence [83], traditional bullying and cyberbullying victimization [27], university students who play video games [94], long-term socialization outcomes [7], children with antisocial tendencies [95], self-determined motivation and well-being [96], academic stress [97], and child behavior problems [83]. Alpha reliability coefficients in the present study were, for academic, 0.853, social, 0.776, emotional, 0.726, family, 0.799, and, for physical, 0.748.

#### 2.3.3. Internalization of Social Values

Internalization of social values was measured with 27 items from the Schwartz (1992) Value Inventory [10], an instrument that has been applied to capture social values internalization through the higher order self-transcendence and conservation values [5,6,7,8,11,35]. Self-transcendence higher order values included universalism (e.g., “A world of beauty (Beauty of nature and the arts)”) and benevolence (e.g., “Honest (Sincere, truthful)”) subscale values. Conservation higher order values included tradition (e.g., “Humble (Modest, going unnoticed)”), conformity (e.g., “Respectful (Showing consideration and honor)”), and security (e.g., “Social order (Social stability)”) subscale values. The participants rated each item using a 99-point scale (depicted which a thermometer), which ranges from one—opposed to my values, to 99—of supreme importance. Modifications (dividing the mean score by ten) were made in each value’s subscale to obtain a score which ranged from 0.10–9.99.

The conservation and self-transcendence higher order values are considered to be oriented to social focus [8,98] and have been used in parenting research as outcomes for social children [5,6,7,35]. Schwartz Value Inventory scales have been applied in multiple research fields, for research as varied as drug use [12,13,99] or well-being across different countries [8]. Cronbach’s alphas for the subscales in present study were universalism, 0.789, benevolence, 0.744, security, 0.852, conformity, 0.628, and tradition, 0.643. These indices were within the range of variation usually observed for these scales [5,6,8].

### 2.4. Data Analysis

To analyze the influence of parenting styles on socialization outcomes, a four-way multifactorial (4 × 3 × 2 × 2) multivariate analysis of variance (MANOVA) was applied to two sets of outcome variables (self-esteem and internalization of values) with parenting styles (authoritative, authoritarian, indulgent, and neglectful), country (Spain, Portugal, and Brazil), age groups (early vs. late adolescents), and adolescent sex (men vs. women) as independent variables. Follow-up univariate *F* tests were conducted for the outcome variables that had multivariate significant overall differences, and significant results on the univariate tests were followed up with Bonferroni comparisons between all possible pairs of means.

## 3. Results

### 3.1. Parenting Style Typologies

Adolescents were classified into one of four parenting typologies: authoritative, indulgent, authoritarian, or neglectful (Table 2). The authoritative group had 586 adolescents (28.0%), with high warmth, M = 3.54, SD = 0.23, and high strictness, M = 2.04, SD = 0.30; the indulgent group contained 463 (22.1%), with high warmth, M = 3.52, SD = 0.24, but low strictness, M = 1.43, SD = 0.19; the authoritarian group had 289 (22.1%), with low warmth, M = 2.82, SD = 0.32, and high strictness, M = 2.01, SD = 0.30; and the neglectful group had 213 (27.7%), with low warmth, M = 2.78, SD = 0.33, and low strictness, M = 1.44, SD = 0.18. Furthermore, analyses also showed that the two parental dimensions, warmth and strictness, consistent with the orthogonality assumption, were modestly inter-correlated, *r* = 0.125, *R*^2^ = 0.02, *p* < 0.01.

### 3.2. Parenting Styles and Self-esteem

The MANOVA for self-esteem yielded statistically significant interaction effects between country and sex, Λ (Lambda de Wilks) = 0.987, *F* (10, 4078.0) = 2.64, *p =* 0.003, country and age, Λ = 0.987, *F* (10, 4078.0) = 2.74, *p =* 0.002, sex and age, Λ = 0.993, *F* (5, 2039.0) = 2.76, *p =* 0.017, country, sex and age, Λ = 0.989, *F* (10, 4078.0) = 2.24, *p =* 0.013, and main effects of parenting, Λ = 0.852, *F* (15, 5629.2) = 22.41, *p* < 0.001, country, Λ = 0.844, *F* (10, 4078.0) = 36.07, *p* < 0.001, sex, Λ = 0.859, *F* (5, 2039.0) = 66.68, *p* < 0.001, and age, Λ = 0.987, *F* (5, 2039.0) = 5.32, *p* < 0.001 (Table 3).

### 3.3. Univariate Effects for Parenting Styles

Indulgent parenting was related to equal or even higher self-esteem than the authoritative style; on the opposite side, authoritarian and neglectful parenting were always related to poor self-esteem outcomes (Table 4). In academic self-esteem, adolescents from indulgent families reported higher scores than their counterparts from authoritative families, whereas those from authoritarian and neglectful homes indicated the lowest scores. In social self-esteem, adolescents who characterized their parents as indulgent and authoritative scored better than their peers from authoritarian and neglectful households. In emotional self-esteem, adolescents with indulgent parents reported higher scores than those with authoritative and authoritarian parents (authoritarian parenting was related to poor emotional self-esteem than authoritative and neglectful styles). In family self-esteem, adolescents with indulgent parents reported higher rates than their counterparts from the other families, adolescents from authoritative homes reported greater scores than those from authoritarian and neglectful households, and the lowest scores corresponded with authoritarian parenting. In physical self-esteem, indulgent parenting was associated with higher scores than authoritarian and neglectful parenting.

### 3.4. Univariate Effects of Demographic Variables

**Figure 1:**
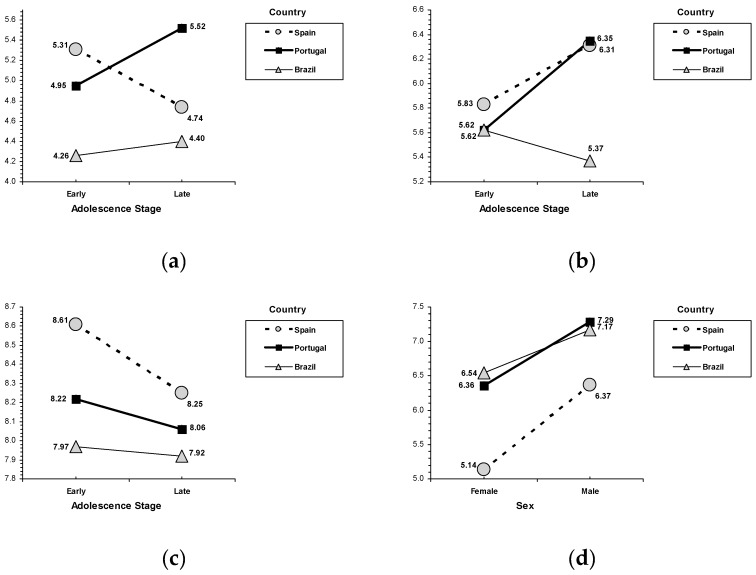
Interaction for sex, age and country, (**a**) emotional self-esteem for females, (**b**) emotional self-esteem for males. Interaction for adolescent stage and country, (**c**) family self-esteem. Interaction for sex and country, (**d**) physical self-esteem.

**Figure 2:**
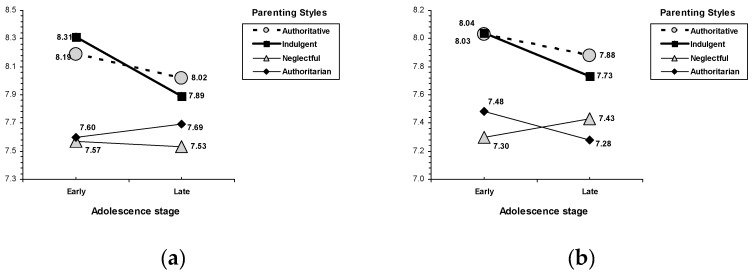
Interactions for parenting style by age. (**a**) universalism, (**b**) security.

In academic self-esteem, the results revealed the main effects for each country, *F* (2, 2043) = 36.76, *p* < 0.001, sex, *F* (2, 2043) = 13.72, *p* < 0.001, and age, *F* (2, 2043) = 14.88, *p* < 0.001. Adolescents from Portugal (M = 6.78, SD = 1.49) and Brazil (M = 6.95, SD = 1.67) reported higher scores than their peers from Spain (M = 6.20, SD = 1.91). Females (M = 6.72, SD = 1.70) reported higher academic self-esteem than males (M = 6.49, SD = 1.77), and early adolescents (M = 6.74, SD = 1.81) indicated higher scores than late adolescents (M = 6.41, SD = 1.60). (ii) In social self-esteem, the results indicated the main effects for each country, *F* (2, 2043) = 12.58, *p* < 0.001. Adolescents from Brazil (M = 8.16, SD = 1.25) reported the highest scores, their peers from Spain indicated the lowest scores (M = 7.79, SD = 1.41), and in the middle position were adolescents from Portugal (M = 7.95, SD = 1.27). (iii) In emotional self-esteem, an interaction effect between sex, age and country was found, *F* (2, 2043) = 7.17, *p* = 0.001 (see Figure 1). For females, the profile in Portugal showed that late adolescents scored higher than early adolescents, whereas, in Spain, emotional self-esteem was higher in early adolescence than in late adolescence, and the lowest emotional self-esteem scores corresponded with both early and late Brazilian adolescents. For males, emotional self-esteem was higher in late adolescence than in early adolescence in Spain and Portugal, although this tendency was not found in Brazil. (iv) In family self-esteem, an interaction effect between age and country was found, *F* (2, 2043) = 3.01, *p* = 0.049 (see Figure 2). Despite the highest scores corresponding with Spain, a decreased general tendency related to age was found only among Spanish adolescents (late adolescents reported less family self-esteem than early adolescents). (v) Regarding physical self-esteem, an interaction effect between sex and country was found, *F* (2, 2043) = 5.13, *p* = 0.006 (see Figure 2). Sex-related differences revealed a similar pattern within each country: males reported more physical self-esteem than females in Spain, Portugal, and Brazil. Additionally, within sex-related differences, scores in Portugal and Brazil were higher than in Spain.

### 3.5. Parenting Styles and Internalization of Values

The MANOVA for internalization of values yielded statistical interaction effects for parenting and sex, Λ = 0.988, *F* (15, 5629.2) = 1.69, *p* = 0.046, country and age, Λ = 0.990, *F* (10, 4078.0) = 2.07, *p* = 0.023, and the main effects of parenting, Λ = 0.927, *F* (15, 5629.2) = 10.47, *p* < 0.001, country, Λ = 0.191, *F* (10, 4078.0) = 17.62, *p* < 0.001, sex, Λ = 0.971, *F* (5, 2039.0) = 12.38, *p* < 0.001, and age, Λ = 0.989, *F* (5, 2039.0) = 4.56, *p* < 0.001 (Table 5).

### 3.6. Univariate Effects for Parenting Styles 

Overall, adolescents from indulgent and authoritative families gave higher priority to self-transcendence values (i.e., universalism and benevolence) and conservation values (security, conformity, and tradition) than their peers from authoritarian and neglectful homes. Additionally, the lowest scores corresponded with those adolescents from neglectful and authoritarian families (Table 6). An interaction effect between parenting style and age was found in universalism, *F* (3, 2043) = 3.42, *p* = 0.017, and security values, *F* (3, 2043) = 3.04, *p* = 0.028 (Figure 2). In a similar way, although some variations within the warmth families (indulgent and authoritative) and lack of warmth families (authoritarian and neglectful) can be drawn from early to late adolescent stage; indulgent and authoritative parenting are related with higher scores in universalism and security values than authoritarian and neglectful styles.

### 3.7. Univariate Main Effects of Demographic Variables 

**Figure 3:**
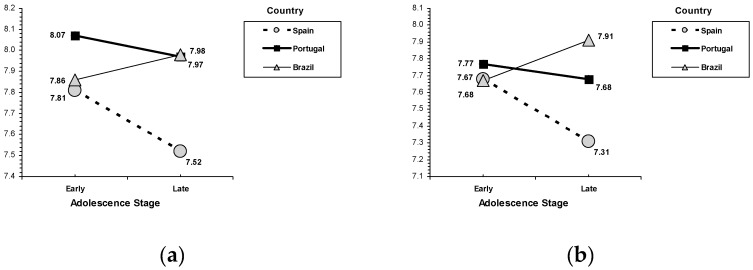

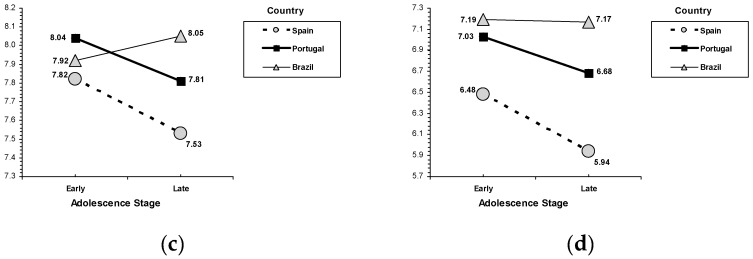
Interactions for adolescent stage and country. (**a**) universalism, (**b**) security, (**c**) conformity, (**d**) tradition.

(i) In self-transcendence values, results revealed the main effects for each country for benevolence, *F* (2, 2043) = 23.52, *p* < 0.001, and for sex in relation to universalism, *F* (2, 2043) = 42.23, *p* < 0.001, and benevolence, *F* (2, 2043) = 36.63, *p* < 0.001. The country-related differences profile revealed that Brazilian adolescents (M = 8.05, SD = 1.27) scored in between the highest scores of Portuguese adolescents (M = 8.33, SD = 1.10) and the lowest scores of Spanish adolescents (M = 7.87, SD = 1.29). Sex-related differences revealed a common pattern in both self-transcendence values: females (M = 8.24, SD = 1.15) reported higher scores in benevolence than males (M = 7.89, SD = 1.30), and universalism scores were greater among females (M = 8.03, SD = 1.18) than among males (M = 7.68, SD = 1.39). Additionally, an interaction effects between age and country was found in universalism, *F* (2, 2043) = 3.93, *p* = 0.020. Whereas weak variations between early and late adolescence were found within Brazilian and Portuguese scores, a generally decreased tendency towards universalism is found only in Spain (scores in late adolescence are lower than in early adolescence, see Figure 3).

(ii) For conservation values, results revealed the main effects for sex in relation to conformity, *F* (2, 2043) = 28.06, *p* < 0.001, and tradition, *F* (2, 2043) = 4.61, *p* = 0.032. Again, sex-related differences showed the highest relation to conservation values for females. In particular, females (M = 8.02, SD = 1.35) reported higher scores in conformity than males (M = 7.69, SD = 1.51), and tradition scores were greater among females (M = 6.79, SD = 1.62) than among males (M = 6.68, SD = 1.60). Also, an interaction effect between age and country was found in security, *F* (2, 2043) = 8.69, *p* < 0.001, conformity, *F* (2, 2043) = 3.293, *p* = 0.037, and tradition, *F* (2, 2043) = 4.05, *p* = 0.018 (Figure 3). A similar country age profile was found in the three conservation values. A general decreased tendency related to age was seen in Spain and, to a lesser extent, in Portugal: late adolescents reported lower scores in security, conformity, and tradition than early adolescents. In Brazil, late adolescents scored equally (in tradition) or even higher (in security and conformity) than early adolescents (see Figure 3).

## 4. Discussion

The results of the study confirm the influence of parenting styles on self-esteem and internalization of values in Spain, Portugal and Brazil. The five self-esteem dimensions, academic, social, emotional, family and physical, are related to the parenting style utilized by the parents. Indulgent parenting emerges as the optimal parenting style for adolescents’ self-esteem, as it is associated with higher adolescent self-esteem than authoritative parenting in academic, emotional, family and physical self-esteem, and no difference can be seen for authoritative parenting on social self-esteem. Authoritarian and neglectful parenting are associated with the poorest levels of self-esteem in the five dimensions. These results are congruent with the new paradigm based on three stages for an optimal parenting style [35], confirming the benefits for adolescent adjustment associated with the third stage (i.e., indulgent style) in the three samples of the study. Furthermore, the results are congruent with previous studies carried out in Spain, Portugal and Brazil from the beginning of the 21st century that have also analyzed the influence of parenting on self-esteem [60,69,100,101,102]. The importance of parental warmth for adolescent self-esteem is evidenced in these results. Indulgent parenting—characterized by the use of parental warmth—is related to higher self-esteem than authoritative parenting—characterized by the use of parental warmth but also by the use of parental strictness—showing that the use of strictness would negatively affect self-esteem. The two parenting styles that are related to low self-esteem are those characterized by the lack of warmth—authoritarian and neglectful parenting.

It is important to note that the effect of parenting on adolescent self-esteem is independent of the differences between self-esteem levels in the three countries and the differences in sex and age. However, the results show some differences in self-esteem depending on the cultural context, which also affects self-esteem differently according to the sex and age of the adolescents, in congruence with the idea that adolescence is not a homogenous life-time period in all cultural contexts [35,103]. In this way, for example, academic self-esteem is higher in adolescents from Brazil and Portugal than in adolescents from Spain. Academic self-esteem is also higher in females and in early adolescents, as previous research has pointed out, suggesting the difference in academic achievement between sex and age that has been identified in other studies [5,104,105]. These results highlight how differences between the three cultural contexts (Spain, Portugal, and Brazil), along with characteristic such as sex and age, can influence adolescents’ self-esteem. However, the relationship between parental practices and the five self-esteem facets is maintained despite these cultural and demographic differences.

Regarding values internalization, the five values priorities—which represent conservation and self-transcendence values—are also related to parenting styles. In the three countries analyzed, adolescents from indulgent and authoritative families present more internalization of universalism, benevolence, security, conformity, and tradition values, than adolescents from authoritarian and neglectful homes. In general, and consistent with previous research [5,6], there is no difference between authoritative and indulgent parenting for values internalization, although there are some small variations between adolescents raised with these two parenting styles in universalism and security values depending on the adolescence stage. In the case of adolescents’ internalization of values, the importance of parental warmth is also evidenced, since the two parenting styles characterized by the use of warmth—indulgent and authoritative—are associated with a higher internalization of values. However, unlike self-esteem, in this case, the use of strictness does not seem to have a negative effect on internalization of self-transcendence and conservation values, since there are no differences between authoritative and indulgent parenting. In contrast, the lack of warmth—which characterizes authoritarian and neglectful parenting—is related with lower internalization of those values.

In addition, the effect of parenting styles over adolescents’ internalization of values is maintained independent of the differences in the internalization of values between the three cultural contexts and the differences by sex and age that are reveled in the results. Differences between the level of the internalization of values appear between the three countries in self-transcendence values, with Portuguese adolescents giving the highest priority to benevolence values, followed by Brazilian adolescents and Spanish in last place. In universalism values, results showed that Spanish adolescents tend to present the lowest internalization, especially in late adolescence. Furthermore, females tend to have higher internalization of universalism and benevolence values than males in the three countries, confirming the results from some previous studies [5,6,7,35]. Some differences are also revealed in conservation values. Again, females give higher priority than males to both conformity and tradition values in the three countries. Moreover, conformity and tradition values tend to be lower in Spain, mainly in late adolescence, where a notable decrease is seen, which also is identified in Portugal.

Present findings add new empirical evidence to recent research that questions the benefits of authoritative parenting (i.e., warmth and strictness) for raising children in all cultural contexts. Although, traditionally, scholars recommended the use of strictness along with warmth as the best parenting strategy, mainly based on research conducted with middle class European–American families [24,36]; present findings suggest that the strictness component might not be necessary or might even have a negative impact on adolescent psychosocial adjustment in the three countries examined (i.e., Spain, Portugal and Brazil), since adolescents from indulgent homes report equal or even higher adjustment than their peers from authoritative households in terms of self-esteem and internalization of social values. The three countries examined (i.e., Spain, Portugal, and Brazil) are usually characterized as horizontal collectivist cultures, in which the self is integrated as a part of the collective (e.g., family) but relationships between members tend to be equalitarian. It is argued that, in these horizontal collectivist cultures, parental imposition and strictness might be negatively perceived by children. The results of the study are in line with some previous studies that have shown that the indulgent style (i.e., warmth without strictness) provides important benefits in terms of psychosocial adjustment for adolescents, including greater school adjustment [106] and optimal learning strategies [97], psychosocial development [95] or environmental empathy and connectedness with nature [84], and protection against alcohol use and abuse [61], marijuana and tobacco [95], personal maladjustment [65] or traditional bullying and cyberbullying victimization [27].

Despite the different pattern for self-esteem and internalization of values among adolescents from Spain, Portugal and Brazil—as revealed the differences by age, sex and country—parents’ practices from the three countries analyzed have a crucial impact during adolescence. However, as previous research has indicated, parents are not the only influence on adolescent development. In this sense, adolescents may also be influenced by different settings inside and outside the family [107,108,109], including family structure (e.g., single parent, both natural parents, or one natural parent and a step-parent) [110], parental employment [111], peers [112], and school [113]. However, despite these influences, a common pattern between parenting styles, self-esteem and internalization of social was found in the three countries: indulgent parenting (parental warmth without parental strictness) is consistently related to the highest levels of adolescent self-esteem and internalization of social values.

Some limitations can be identified in the study regarding the strength of analyzing parenting styles across different cultural contexts. The study was cross-sectional and conclusions about directionality are only based on the previous literature on parenting research. Furthermore, adolescents report their own and their parents’ behavior, despite the fact that it has been shown that adolescents’ reports show lower social desirability than parents’ reports (e.g., child reports of parenting practices were significantly correlated with a greater number of psychosocial indicators than parent reports) [114]. Finally, a common pattern of invariance was guaranteed, allowing us to emphasize a common pattern between parenting and adolescents’ self-esteem and values internalization in Spain, Portugal, and Brazil.

## 5. Conclusions

The present study adds new evidence to the literature debate about the best parenting, using a three-stage conceptual framework in order to identify which of the three stages (i.e., first, second or third) is optimal for the three different countries in the current society. Our findings indicated the benefits of adolescent self-esteem and internalization of values associated with the third parenting style (i.e., indulgent style) in Brazil, Spain and Portugal. Results revealed sex-and age-related differences as well as differences by country in the adjustment criteria examined (i.e., self-esteem and internalization of values) among Brazilian, Spanish and Portuguese adolescents. Nevertheless, the influence of parenting over adolescent self-esteem and values internalization is maintained independent of the differences in self-esteem and value value priorities observed depending on the cultural context, the sex and age of the adolescents. Only indulgent parenting (use of warmth) is consistently associated with the highest adjustment in terms of self-esteem and internalization of social values. Future research should be carried out to examine parental socialization across the world, comparing different countries, ethnic and cultural contexts and using different adjustment criteria, in order to identify, for each cultural context, the best parental strategy to promote child and adolescent development.

## Figures and Tables

**Table 1:** Distribution of sample by sex, adolescent stage and country.

Variables		Frequency	Percent	Age		
				Range	Mean	SD
**Sex**						
Women		1090	52.1	12–18	14.30	1.76
Men		1001	47.9	12–18	14.25	1.71
Adolescence stage						
Early		1267	60.6	12–14	13.07	0.91
Late		824	39.4	15–18	16.14	0.82
Sample		2091	100.0	12–18	14.28	1.74
Country						
Spain	Sex					
	Women	414	56.4	12–18	14.57	1.76
	Men	379	43.6	12–18	14.36	1.68
	Adolescence stage					
	Early	443	55.9	12–14	13.15	0.92
	Late	350	44.1	15–18	16.15	0.82
	Sub-sample	793	37.9	12–18	14.47	1.73
Portugal	Sex					
	Women	381	56.4	12–18	14.09	1.75
	Men	294	43.6	12–18	14.08	1.81
	Adolescence stage				
	Early	445	65.9	12–14	12.99	0.95
	Late	230	34.1	15–18	16.20	0.84
	Sub-sample	675	32.3	12–18	14.08	1.78
Brazil	Sex					
	Women	295	47.4	12–18	14.19	1.73
	Men	328	52.6	12–18	14.27	1.64
	Adolescence stage					
	Early	379	60.8	12–14	13.06	0.85
	Late	244	39.2	15–18	16.06	0.8
	Sub-sample	623	29.8	12–18	14.23	1.68
Sample		2091	100.0	12–18	14.28	1.74

**Table 2:** Distribution of parenting style groups, mean scores, and standard deviations on main measures of parental dimensions.

	Total	Authoritative	Indulgent	Authoritarian	Neglectful
Frequency	2091	586	463	462	580
Percent	100	28.0	22.1	22.1	27.7
Warmth					
Mean	3.17	3.54	3.52	2.82	2.78
SD	0.46	0.23	0.24	0.32	0.33
Strictness					
Mean	1.73	2.04	1.43	2.01	1.44
SD	0.39	0.30	0.19	0.30	0.18

**Table 3:** Multivariate analysis of variance (MANOVA) factorial (4 ^a^ × 2 ^b^ × 2 ^c^ × 2 ^d^) for self-esteem.

Source of Variation	Λ	*F*	df_between_	df_error_	*p*
(A) Parenting Style	0.852	22.41	15	5629.2	<0.001
(B) Country	0.844	36.07	10	4078.0	<0.001
(C) Sex	0.859	66.68	5	2039.0	<0.001
(D) Age	0.987	5.32	5	2039.0	<0.001
A × B	0.983	1.17	30	8158.0	0.235
A × C	0.994	0.87	15	5629.2	0.599
A × D	0.994	0.78	15	5629.2	0.705
B × C	0.987	2.64	10	4078.0	0.003
B × D	0.987	2.74	10	4078.0	0.002
C × D	0.993	2.76	5	2039.0	0.017
A × B × C	0.981	1.32	30	8158.0	0.112
A × B × D	0.982	1.27	30	8158.0	0.147
A × C × D	0.992	1.03	15	5629.2	0.415
B × C × D	0.989	2.24	10	4078.0	0.013
A × B × C × D	0.990	0.68	30	8158.0	0.902

^a^*a*_1_, authoritative; *a*_2_, indulgent; *a*_3_, authoritarian; *a*_4_, neglectful; ^b^ b_1_, Spain; b_2_, Portugal; b_3_, Brazil; ^c^ *c*_1_, female; *c*_2_, males; ^d^*d*_1_, 12–14 years old; *d*_2_, 15–17 years old; df—degrees of freedom.

**Table 4:** Means and (standard deviations) of parenting style, and main univariate *F* values for self-esteem.

Self-Esteem	Authoritative	Indulgent	Authoritarian	Neglectful	*F*(3, 2043)
Academic	6.79 ^2^	7.07 ^1^	6.26 ^3^	6.34 ^3^	18.82 ***
	(1.69)	(1.58)	(1.85)	(1.72)	
Social	8.10 ^1^	8.22 ^1^	7.68 ^2^	7.81 ^2^	17.11 ***
	(1.27)	(1.21)	(1.40)	(1.36)	
Emotional	5.24 ^2, a^	5.64 ^1^	4.92 ^2, b^	5.49 ^a^	12.88 ***
	(1.92)	(2.01)	(1.94)	(1.94)	
Family	8.58 ^2^	8.90 ^1^	7.32 ^4^	7.99 ^3^	100.83 ***
	(1.15)	(1.04)	(1.88)	(1.55)	
Physical	6.49	6.71 ^1^	6.21 ^2^	6.22 ^2^	7.43 ***
	(1.92)	(1.80)	(1.91)	(1.86)	

Note: Bonferroni test; α = 0.05; 1 > 2 > 3; a > b; *p* < 0.05, *p* < 0.01, *** *p* < 0.001.

**Table 5:** MANOVA Factorial (4 ^a^ × 2 ^b^ × 2 ^c^ × 2 ^d^) for value priorities.

Source of variation	Λ	*F*	df_between_	df_error_	*p*
(A) Parenting Style	0.927	10.47	15	5629.2	<0.001
(B) Country	0.919	17.62	10	4078.0	<0.001
(C) Sex	0.971	12.38	5	2039.0	<0.001
(D) Age	0.989	4.56	5	2039.0	<0.001
A × B	0.981	1.33	30	8158.0	0.106
A × C	0.995	0.65	15	5629.2	0.839
A × D	0.988	1.69	15	5629.2	0.046
B × C	0.995	1.11	10	4078.0	0.350
B × D	0.990	2.07	10	4078.0	0.023
C × D	0.999	0.44	5	2039.0	0.820
A × B × C	0.980	1.36	30	8158.0	0.091
A × B × D	0.983	1.20	30	8158.0	0.206
A × C × D	0.993	0.93	15	5629.2	0.534
B × C × D	0.998	0.44	10	4078.0	0.926
A × B × C × D	0.989	0.75	30	8158.0	0.838

^a^*a*_1_, authoritative; *a*_2_, indulgent; *a*_3_, authoritarian; *a*_4_, neglectful. ^b^ b_1_, Spain; b_2_, Portugal; b_3_, Brazil. ^c^ *c*_1_, female; *c*_2_, males. ^d^ *d*_1_, 12–14 years old; *d*_2_, 15–17 years old.; df—degrees of freedom.

**Table 6:** Means and (standard deviations) of parenting style, and main univariate *F* values for value priorities.

Values	Parenting Style				
	Authoritative	Indulgent	Authoritarian	Neglectful	*F*(3, 2043)
Self-transcendence					
Universalism	8.12 ^1^	8.15 ^1^	7.63 ^2^	7.55 ^2^	28.43 ***
	(1.15)	(1.14)	(1.40)	(1.37)	
Benevolence	8.23 ^1^	8.35 ^1^	7.89 ^2^	7.86 ^2^	15.82 ***
	(1.19)	(1.15)	(1.30)	(1.25)	
Conservation					
Security	7.97 ^1^	7.92 ^1^	7.41 ^2^	7.35 ^2^	28.28 ***
	(1.19)	(1.20)	(1.45)	(1.41)	
Conformity	8.18 ^1^	8.21 ^1^	7.48 ^2^	7.57 ^2^	36.49 ***
	(1.29)	(1.26)	(1.60)	(1.44)	
Tradition	7.04 ^1^	6.98 ^1^	6.54 ^2^	6.39 ^2^	22.56 ***
	(1.56)	(1.59)	(1.59)	(1.60)	

Note: Bonferroni Test. α = 0.05; 1 > 2; a > b; *p* < 0.05, *p* < 0.01, *** *p* < 0.001.
